# Digital Health Interventions for Falls Prevention in Older People

**DOI:** 10.1007/s11914-026-00992-z

**Published:** 2026-10-26

**Authors:** Kim Delbaere, Meghan Ambrens, Mei Ling Lim, Paulo Pelicioni, Kimberley S. van Schooten

**Affiliations:** 1 School of Health Sciences, University of New South Wales, Kensington, NSW Australia; 2 Falls, Balance and Injury Research Centre, Neuroscience Research Australia, Randwick, NSW Australia; 3 The George Institute of Global Health, Randwick, NSW Australia; 4 School of Population Health, University of New South Wales, Kensington, NSW Australia

**Keywords:** Accidental falls, Older people, Digital health, Technology, Health service delivery

## Abstract

**Purpose of Review:**

Implementation of falls prevention programs at scale remains challenging due to resource constraints, clinic dependency and inequitable access. We critically review digital health technologies and map these to the World Falls Guidelines (WFG) risk-stratified pathway, from risk screening to intervention delivery, appraising the evidence, clinical readiness and implementation challenges.

**Recent Findings:**

Self-managed digital exercise programs have the strongest evidence base, with three trials demonstrating reductions in falls among community-living older people and economic data supporting cost-effectiveness in those with a history of falls. Other technologies demonstrate feasibility, but none has demonstrated falls reduction in adequately powered trials. Implementation evidence is emerging but concentrated in high-income, digitally literate populations.

**Summary:**

Mapped to the WFG pathway, digital exercise programs are ready for routine implementation; realising broader potential requires regulatory frameworks that set evidence standards for digital products, funding models that support digital care and sustained commitment to equitable access across the diversity of older people.

## Introduction

**Fig. 1:**
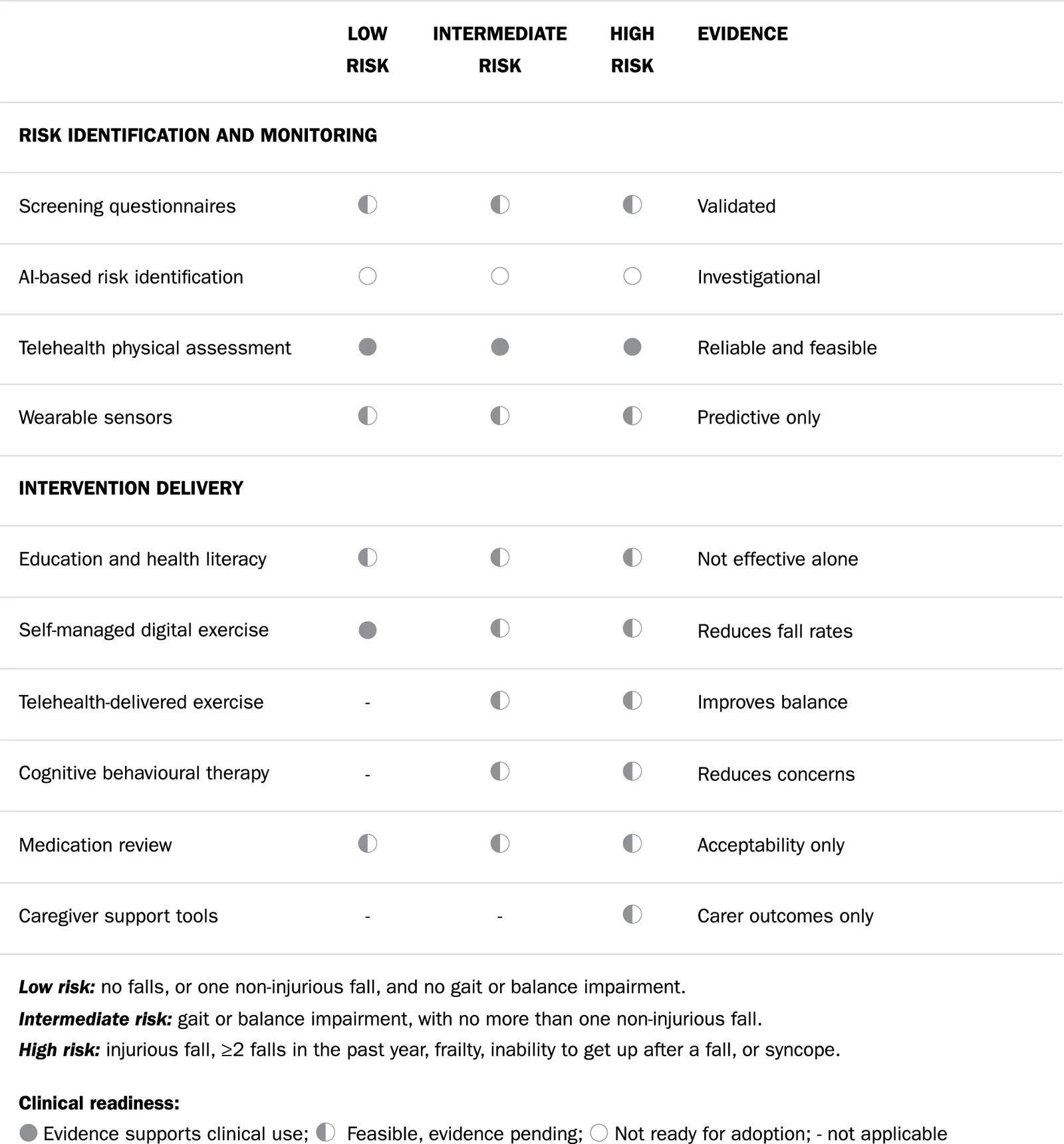
Clinical readiness of digital falls prevention technologies across the World Falls Guidelines pathway

Falls prevention is central to maintaining mobility, quality of life and independence as people age. [1] Yet despite three decades of evidence, implementation at scale remains the defining challenge. Many effective falls prevention programs are resource-intensive, clinic-dependent and difficult to deliver equitably at scale in an ageing population. Digital health technologies increasingly create opportunities to address this gap. Several randomised controlled trials have evaluated the effect of digital health interventions on falls outcomes, and the evidence base is growing rapidly. Furthermore, in 2025, 97% of Australians aged 65–74 and 88% of those aged 75 + used a mobile phone to access the internet (compared to 18% in 2017) [2]. The critical question is therefore no longer whether digital technologies are feasible or acceptable to older people [3] but whether there is evidence that digital technologies can improve access to evidence-based care and, ultimately, reduce falls at the population level. This narrative review is structured around the World Falls Guidelines (WFG) risk-stratified falls-prevention pathway, which begins by identifying who is at risk and matches intervention intensity to falls risk across the full continuum of care [1]. We map digital health technologies from risk identification and monitoring through to intervention delivery and implementation considerations, while appraising the strength of the evidence and readiness for clinical use, as summarised in Fig. 1.

### Risk Identification and Monitoring

Screening and monitoring span all three WFG risk levels. Screening questionnaires and artificial intelligence (AI) serve as the entry point to the pathway, while mobility assessment, potentially via wearable sensors or telehealth, differentiates low, intermediate and high risk; and ongoing monitoring supports adaptive risk management across the pathway.

### Screening Questionnaires

Systematic falls risk screening in routine clinical and community settings remains inconsistent, yet it is the entry point to the WFG pathway. The WFG recommends the three-key questions (3KQ) and Stay Independent questionnaire as brief, validated tools for population-level screening: both are question-only instruments with demonstrated predictive validity in community-dwelling older people [1]. A precedent for their digital integration exists in the CDC’s falls prevention framework, *STEADI*, which successfully embedded the 3KQ into Electronic Health Record (EHR)-based clinical decision support tools across US primary care settings [4]. Digital screening alone is unlikely to improve outcomes unless embedded within clear referral pathways that ensure people identified as at risk receive evidence-based interventions. Future trials linking screening to referral uptake and falls outcomes are still needed to establish effectiveness.

### The Emerging Role of Artificial Intelligence

AI cuts across the falls prevention pathway, supporting both risk identification and clinical decision-making. Most current approaches involve machine learning models that use demographic, clinical, mobility and sensor-derived data to identify people at high risk of falls [5]. Examples include the use of large language models to identify topic changes in clinical notes (or EHR) as early predictors of falls, [6, 7] which could passively detect fall-related content and trigger automated screening prompts without adding burden. AI-driven decision support systems may help prioritise referrals, identify high-risk medication combinations, or tailor intervention recommendations according to individual risk profiles. Emerging digital platforms are integrating screening, monitoring, and exercise prescription into a single system that supports both clinicians and patients [8]. However, the evidence base is still developing and is often based on relatively small or homogeneous datasets that lack external validation. AI scribes and natural language processing (NLP)-based screening tools are technically feasible but have not been evaluated in falls prevention. Further research must address external validation, algorithmic transparency, bias and health system integration before clinical adoption can be recommended.

### Telehealth-Based Physical Assessment

Telehealth physical assessment extends access to people who are unable or unwilling to attend clinics and can support ongoing review within integrated models of care. Established tests, including the Timed Up and Go, Short Physical Performance Battery, 5-times Sit-to-Stand and 4-m walk, demonstrate adequate-to-good reliability when tested remotely and by different raters [9–11], including older people with a history of falls [12], Parkinson’s disease [13] and dementia [14], with poor reliability of 4-m walking speed [10, 12, 14]. Feasibility is supported across populations, [9–15] though digital literacy, access to technology and connectivity remain barriers to broader participation. Safety requires careful participant selection. High falls risk, severe cognitive impairment, concerns about falling and unsuitable home environments should prompt additional safety measures [11]. Overall, telehealth assessments are reliable, feasible and safe across a range of populations and are ready for clinical use with appropriate safety protocols. Trials are needed to determine whether increased access improves risk stratification or reduces falls compared to standard practice remains unknown.

### Wearable Sensors for Fall Risk Assessment and Monitoring

Wearable technologies, including smartwatches, fitness trackers, personal emergency response systems (PERS) and modern hearing aids, are increasingly part of the daily lives of older people, with many now incorporating motion sensors capable of monitoring mobility and falls risk. Falls risk is dynamic,^16^ influenced by fluctuations in health, function, behaviour and the environment [17]. Daily-life gait characteristics consistently predict future falls in community-living older people. Measures such as gait variability, gait regularity, harmonicity and entropy have been linked to falls risk, [18] often outperforming traditional clinical measures alone [19, 20]. Longitudinal monitoring may enable earlier detection of functional decline, responses to intervention [21], or transient periods of risk associated with illness, medication changes or reduced activity. Emerging work suggests that adaptive falls prevention, in which intervention intensity varies with real-time risk status, may be supported by continuous monitoring [22]. However, several challenges remain before wearable monitoring can be routinely implemented into clinical care [23]. Prediction algorithms require external validation across populations and devices. Questions remain regarding optimal monitoring protocols, clinically meaningful thresholds and interoperability with health systems. As highlighted in the WFG, wearable monitoring should be viewed as complementary rather than a replacement for clinical assessment. Its clinical value will depend not on predictive accuracy alone, but on whether sensor-driven changes trigger effective interventions that reduce falls.

### Intervention Delivery

Digital technologies offer new delivery modes for falls prevention interventions with an established evidence base. This section examines six intervention domains: education and health literacy, which applies across all risk levels; self-managed digital exercise and telehealth-delivered exercise, mapped to low and intermediate risk; cognitive behavioural therapy for psychological barriers to engagement; medication review supported by clinical decision tools; and caregiver-supported approaches for people at highest risk.

### Education and Falls Health Literacy

Health literacy shapes how older people understand falls risk, interpret clinical advice and apply preventive strategies in daily life [24]. Adequate health literacy supports informed decision-making, strengthens self-efficacy and can reduce concern about falling, a known barrier to physical activity and other preventive behaviours [25]. Among health literacy domains, self-efficacy has been identified as most critical for sustaining engagement in physical activity [26]. Targeted education that improves knowledge, corrects misconceptions and builds confidence is one approach to strengthening health literacy. At a population level, digital health promotion campaigns offer scalable reach, but evidence for their effectiveness in fall outcomes remains limited [27]. The WFG recommends education for all older people regardless of risk level [28]. However, education alone does not reduce falls. Digital platforms are most valuable when education is embedded within broader interventions that simultaneously address balance, motivation and opportunity for behaviour change.

### Self-Managed Digital Exercise Programs

Self-managed digital exercise programs can reduce falls among lower-risk older people living in the community, as demonstrated through three published RCTs since 2021. *StandingTall* is an app-based program of progressive, individually tailored balance, stepping and functional strength exercises, with the recommended dose of 120 min/week for 24 months. [29] *Smart* ± *Step* is a TV-connected exergame in which participants step on a mat to play games requiring rapid, accurate stepping and decision-making, with the recommended dose of 120 min/week for 12 months. [30] Safe Step is a self-managed, unsupervised app-based program of 10 progressive strength and balance exercises, with a recommended dose of 90 min/week for 12 months. [31] The *StandingTall* trial (n = 503, ≥ 70 years), using a balance exercise app, reduced fall rate by 16% (IRR 0.84, 95% CI 0.72–0.98) and injurious fall rate by 20% (IRR 0.80, 95% CI 0.66–0.98) over 24 months. [29] The *Smart* ± *Step* exergaming trial (n = 769, ≥ 65 years) similarly reduced falls over 12 months (IRR 0.74, 95% CI 0.56–0.98) [30]. *Safe Step* (n = 1,628, ≥ 70 years) showed no significant reduction in fall rate but a modest reduction in the proportion of people experiencing at least one fall (53% vs 60%; RR 0.89, 95% CI 0.80–0.99) [31]. A review of 10 digitally delivered *Otago* exercise programs found improvements in balance, strength and fall efficacy, [32] suggesting potential pathways through which these programs may influence fall risk. Furthermore, systematic review evidence supports feasibility, safety and acceptability across programs. [33]

For people at higher falls risk, including those over 80, or living with Parkinson’s disease, dementia or mild cognitive impairment, evidence is more limited and generally of lower quality [34]. A systematic review of nine trials looking at the effectiveness of exergames (1,385 participants aged ≥ 60 years at higher falls risk) found lower fall rates compared to usual care (IRR 0.53, 95% CI 0.41–0.68) with moderate-certainty evidence for reduction in fallers at 12 months (RR 0.75, 95% CI 0.62–0.92) [35]. Notably, an economic evaluation of *StandingTall* found the program was both less costly and more effective than usual care in people with a history of falls, suggesting particular value at the intermediate risk level of the WFG pathway [36]. *StandingTall*, with added cognitive-motor exercises, is currently being evaluated in a randomised controlled trial in 518 higher-risk older people. [37]

Self-managed digital exercise programs are ready for clinical implementation at the lower-risk level of the WFG pathway, with a small but consistent evidence base. Evidence for intermediate and high-risk populations remains limited and should not yet drive routine adoption. Co-design with older people, as demonstrated by both *StandingTall* and KOKU, is a key feature of effective digital programs: iterative end-user involvement in development produces programs that are usable, accessible and aligned with the needs of older people [38, 39]. The key implementation challenge is sustaining engagement without clinician support: a secondary analysis of pooled data from both *StandingTall* trials identified eight distinct adherence trajectories over 52 weeks, demonstrating that adherence is a dynamic behaviour shaped by cognitive and physical capability rather than age or general health, findings that should inform the design of adaptive engagement strategies in future programs. [40]

### Telehealth-Delivered Exercise Programs

Telehealth occupies the space between fully self-managed digital interventions and traditional face-to-face delivery, by combining remote exercise delivery with real-time clinician support for feedback, progression and safety oversight. Systematic review evidence suggests that telehealth-delivered exercise can improve balance and reduce falls risk in community-living older people, with effects comparable to face-to-face exercise, though evidence for falls prevention remains uncertain [41]. Recent trials have extended telehealth exercise delivery to pre-frail and frailer populations, including those with chronic conditions, previous falls, neurological conditions and people living in long-term care [35, 42–46]. Improvements in strength, mobility and balance have been reported consistently, with good acceptability and adherence. However, interventions vary substantially in frequency, duration, level of clinician support and target population. When telehealth was compared to in-person delivery, in-person delivery showed better adherence and greater improvements in motor function, including balance and gait [45]. A cluster RCT of physiotherapist-led telehealth coaching reported improvements in daily step count, physical activity, walking duration and wellbeing, but no significant effect on falls at 12 months. [47] This suggests telehealth could be best positioned as an accessible alternative for those unable to attend in-person services, rather than as a preferred mode of delivery. Telehealth exercise is feasible and safe across a range of populations and can be offered as an alternative where in-person access is a barrier, although evidence for falls reduction is still pending. The key challenge is implementation at scale: understanding real-world uptake, workforce requirements and optimal models of clinician support in routine care. [42]

### Cognitive Behavioural Therapy

Concerns about falling are highly prevalent among older people and is a key modifiable risk factor for activity avoidance, functional decline, and subsequent falls [48, 49]. Cognitive behavioural therapy (CBT), alone or combined with exercise, has strong evidence for reducing concerns about falling by addressing maladaptive beliefs and promoting adaptive coping and graded re-engagement in daily activities [28, 50]. Digital delivery extends the reach of CBT to older people with access barriers. A pilot RCT of a 6-week self-guided online CBT program demonstrated high adherence and low attrition in older people [51]. The Own Your Balance trial, combining an 8-week online CBT program with digital exercise and telehealth motivational interviewing to reduce concerns about falling, is currently underway [52]. Digital CBT to address concerns about falling is feasible and acceptable, supported by a strong face-to-face evidence base and sound theoretical rationale. Falls outcome data from adequately powered trials are pending, and clinical adoption should await those results.

### Medication Review

Falls-risk-increasing drugs (FRIDs) are a significant and modifiable contributor to falls risk, and medication review is recommended across all WFG risk levels. STOPPFall, a validated FRID screening tool, was developed to harmonise clinical practice and guidelines across Europe [53]. Building on this, the ADFICE_IT clinical decision support system provides personalised deprescribing advice through a shared decision-making interface for clinicians and patients [54, 55]. Clinician evaluation found ADFICE_IT acceptable for fast-paced clinical environments, with actionable recommendations, transparent risk information and visualisation of individual falls risk estimates [56]. A multicentre trial is currently underway; no results are yet available. Digital medication review tools are in the early stages of evaluation. The evidence base currently comprises data on tool development and clinician acceptability. Clinical adoption should await trial results demonstrating effect on medication-related falls.

### Supporting Caregivers and Support Persons

Caregivers and support persons play an important role in falls prevention for older people at highest risk, particularly those with cognitive impairment or complex care needs. Digital tools have been developed to support caregivers both in delivering falls prevention and in managing their own health and wellbeing. An app that helps caregivers and older people identify fall hazards in the home environment reduced the risk of accidents and improved preventive practices [57]. Two digital general physical activity programs for carers of people with dementia found good acceptability but low adherence, with limited time and age-related technical difficulties cited as common barriers [58, 59]. The evidence in this area is limited and largely indirect, with most studies focusing on carer wellbeing and physical activity rather than on fall outcomes in the person they support. Digital tools for caregiver support are acceptable in principle but require further development and evaluation before their contribution to falls prevention can be determined.

### Implementation Considerations

The implementation of digital falls prevention across the WFG pathway requires understanding the barriers and enablers that shape adoption, older people’s preferences, the economic case for investment and the equity implications of digital delivery.

### Barriers and Enablers

Barriers and enablers to implementation operate at the individual, organisational and system level. At the individual level, health and digital literacy, age-related attitudes toward technology and low confidence in using digital devices are common barriers for both older people and clinicians [60, 61]. Clinicians’ reluctance to prescribe digital programs, and unfamiliarity with the evidence base further limit uptake. Enablers include targeted education and training for clinicians and end users, as well as identifying local champions to promote adoption [62]. At the organisational level, limited infrastructure, including restricted internet access, device availability and IT funding, constrains implementation, particularly in aged care and rural settings [60, 61]. Integration with existing workflows and electronic medical records is a practical barrier, as is competition with other organisational priorities. Enablers include program adaptability to local contexts, stakeholder engagement and development of formal implementation plans. At the system level, alignment with national policy and reimbursement frameworks is a key driver of adoption; without clear funding pathways, digital programs remain demonstration projects rather than routine services. Building coalitions across health, aged care and technology sectors, and establishing sustainable financial models, are essential system-level enablers. [63, 64]

### End-User Preferences

Older people’s preferences for falls prevention programs are important to successful digital implementation. A discrete choice experiment (DCE) found that 82% of older people did not consider participation in exercise-based programs worthwhile even where the program was shown to eliminate falls risk entirely, [65] underscoring that effectiveness alone does not drive uptake. A DCE of an eHealth falls prevention program identified cost, mode of delivery, class size, personalisation, program duration, and access to professional support as important preferences [66]. Digital programs can address several of these. They offer flexibility in when and where exercise is accessed, allow users to control session duration, and enable tailoring that becomes increasingly important with age [3, 38]. However, older people also value socially engaging formats such as group-based exercise for motivation and perseverance [3, 66]. Hybrid models that combine digital delivery with optional group or peer components may therefore better reflect the range of preferences held by older people. Understanding and incorporating patient preferences is essential for uptake, yet remains underdeveloped in the digital falls prevention literature. Some studies have incorporated preferences into intervention design, [38, 39] but future trials should routinely elicit preferences prior to program design.

### Economic Evidence for Digital Falls Prevention

A systematic review of falls prevention programs for community-living older people (21 economic evaluations, 6 costing studies) found results ranging from dominant interventions to an ICER of US$279,802/QALY; 17 of 24 were considered cost-effective at a US$100,000/QALY threshold (moderate certainty) [67]. Older people and those at high falls risk offered the greatest value for money, while unsupervised exercise offered relatively less [67]. *StandingTall* is the only digital falls prevention program with a published economic evaluation, with a base-case ICER of $58,039/QALY within Australia's willingness-to-pay threshold, and an incremental cost of $4,785 per fall prevented [36]. Consistent with the broader literature, *StandingTall* dominated usual care in older people with a history of falls (i.e., higher risk) [36]. Notably, despite being unsupervised, *StandingTall* remained cost-effective at the base case, suggesting digital delivery may partly offset the value disadvantage typically associated with unsupervised exercise [36]. Early evidence suggests that digital exercise programs can be cost-effective, though the economic evidence base remains narrow and does not yet extend across the full falls prevention pathway.

### Equity and Reach

Recent evidence suggests that only 18% of overall telehealth physiotherapy trials address health equity [68]. Inequity in access to digital falls prevention arises from multiple factors, including social determinants of health, financial constraints, limited access to technology and mobility restrictions [69]. A DCE of Aboriginal and Torres Strait Islander preferences for falls prevention programs found that culturally specific services and lower out-of-pocket costs were the main preferences for accessing care, highlighting that equity requires more than access to technology [70]. Digital exclusion extends beyond internet access and device ownership. Vision impairment, hearing impairment, cognitive impairment, language barriers, financial constraints and low digital confidence may all affect an older person’s ability to engage with digital fall prevention initiatives. Future implementation studies should routinely evaluate whether interventions reduce or inadvertently widen health inequities.

### Clinical and Research Implications

Digital health technologies offer new delivery modes for evidence-based falls prevention, but clinical readiness varies considerably across the WFG pathway. This review maps that readiness, providing health systems with a structure for identifying where digital technology is ready for implementation, where further evidence is needed and what gaps remain. Digital solutions should be judged not by their novelty but by whether they reduce falls, improve health equity and help close the implementation gap, the same standard applied to any clinical intervention.

### What is Ready for Clinical Practice?

Exercise is the only domain with direct trial evidence of falls reduction and is ready for implementation. Three RCTs have demonstrated that self-managed digital balance programs reduce fall rates in community-living older people, [29–31] well aligned with the low-risk WFG pathway where most older people can self-manage their risk with appropriate guidance. Evidence is emerging at the intermediate-risk level, with economic data from *StandingTall* suggesting particular value in those with a history of falls [36]. Digital exercise programs are suitable for implementation into health and aged-care services now, ideally with periodic telehealth or face-to-face contact to support engagement and tailor progression [62]. Telehealth-guided exercise extends this approach to those requiring closer clinical supervision and is a feasible alternative where in-person access is a barrier, though its effect on falls remains uncertain. [42]

### What Requires Further Evidence Before Clinical Adoption?

For risk identification and monitoring, the evidence base is in its early stages and clinical caution is warranted. Wearable monitoring shows both potential and limitations [18]. Free-living gait measures consistently predict falls [19–21] and are increasingly accepted by clinicians [23]. Nonetheless, routine adoption will still depend on external validation in diverse populations, clinically meaningful thresholds, reliable data accuracy across consumer-grade devices, and implementation pathways within EHRs. AI-based decision-support tools have been developed using small or demographically narrow datasets and require prospective validation, algorithmic transparency and bias assessment before clinical use. Digital implementation of the 3KQ and telehealth-based physical assessment show strong face validity and logistical feasibility and are ready as clinical tools, [10] but neither has been shown to improve falls outcomes compared with standard practice.

For interventions beyond digital exercise programs, the evidence base is still largely emerging. Digitally delivered CBT has been shown to reduce concern about falling, [51] with falls outcomes from adequately powered trials underway [52]. Coaching via telehealth, using motivational interviewing, is feasible but has not yet demonstrated an effect on falls at 12 months. [47] Medication review tools, such as STOPPFall, offer a structured approach to deprescribing fall-risk-increasing drugs, but evidence of fall reduction from their digital deployment in clinical practice is lacking [53]. Digital caregiver support tools are acceptable in principle, but their contribution to falls prevention remains limited and largely indirect. Population-based digital falls health literacy campaigns share the same limitation as their non-digital predecessors: awareness alone does not reduce falls without accessible, funded services. [27]

### Research Priorities

The most urgent research need is adequately powered trials with falls as a primary outcome. Several research areas, including digitally delivered CBT, motivational coaching and digital medication review, have established feasibility but lack the trial evidence needed to justify clinical adoption. For exercise, head-to-head trials comparing digital and in-person delivery are needed, alongside trials in higher-risk groups, including people living with dementia, Parkinson’s disease or frailty, to evaluate safety, efficacy and tailoring. Secondly, multifactorial interventions are recommended for high-risk older people but consistently difficult to deliver in practice. People rarely receive the right combination of interventions for their individual risk profile, and effectiveness is often reduced by selective engagement with recommended components such as exercise [71]. Digital platforms offer a genuine opportunity to coordinate adaptive, personalised, multicomponent care in ways that traditional clinic-based programs cannot. Testing this potential is a priority for the next decade, which directly leads to the third priority: implementation research. Demonstrating efficacy in controlled settings is necessary but insufficient; understanding how digital falls prevention can be adopted into routine health and aged-care services remains a critical gap. Fourth, the digital falls prevention literature is almost entirely drawn from high-income countries; evidence from low- to middle-income countries, where specialist services are scarce and infrastructure barriers are more pronounced, remains a critical and largely unaddressed gap. Fifth, equity must be an explicit design priority: current trial samples are overwhelmingly community-living, digitally literate, English-speaking older people, and generalisability to those with lower digital literacy, limited English proficiency, cultural and linguistic diversity or socioeconomic disadvantage cannot be assumed. Finally, economic evaluation should accompany all efficacy trials; the only published data for digital exercise suggest cost-effectiveness, [36] but evidence from other intervention domains and higher-risk populations is lacking.

### Health Systems, Regulatory and Policy Priorities

Translating digital falls prevention from research into routine care requires systemic action across evidence standards, regulation, implementation, equity and data infrastructure. The abundance of commercial apps without rigorous evaluation represents a risk to individuals and the field; wearables and AI compound this through proprietary algorithms that limit accountability and independent validation. In most countries, including Australia, digital falls prevention programs are classified as wellness products and fall entirely outside therapeutic goods regulation. There is no alternative framework to fill the gap, and clinicians cannot prescribe digital technologies. In contrast, Germany’s DiGA scheme enables prescribing of certified, reimbursed apps, creating an incentive for evidence-based apps. The persistent implementation gap reflects structural barriers, as demonstrated by workforce shortages, fragmented services and fee-for-service funding models that do not support ongoing digital care, which technology alone cannot resolve. Equitable access requires subsidised devices, culturally adapted resources and supported onboarding. Finally, a linked national data infrastructure that connects health, aged-care and digital program records is needed to evaluate population-level impact and guide resource allocation.

## Conclusions

Digital health offers a genuine opportunity to expand access to effective falls prevention at population scale. However, realising that potential depends on matching technology to evidence. Mapped against the WFG pathway, the evidence shows that self-managed digital exercise programs are ready for implementation now; telehealth-guided exercise is a feasible alternative for those with access barriers; and several other applications (e.g., wearable monitoring, AI-based decision support, digitally delivered CBT and medication review) show promise but require adequately powered trials before clinical adoption can be recommended. The defining challenge for the next decade is embedding those new technologies with proven efficacy into health systems equitably, sustainably and at scale. Achieving this will require regulatory frameworks that protect consumers, funding models that support digital care, and a sustained commitment to evaluating reach and impact across the full diversity of older people.

## Key References

- Pinheiro MB et al. Economic evaluations of fall prevention exercise programs: a systematic review. Br J Sports Med 2022;56:1353–65
  - ○ This systematic review synthesises 21 economic evaluations and 6 costing studies of falls prevention exercise programs for community-dwelling older people, providing the most comprehensive economic evidence base in the field. Critically, programs targeting people at higher risk of falls consistently demonstrated the greatest economic value.
- Lewis SR et al. Population-based interventions for preventing falls and fall-related injuries in older people. Cochrane Database Syst Rev 2024;(1).
  - ○ This Cochrane systematic review provides the highest-level evidence synthesis of population-based falls prevention, at which digital technologies offer the greatest potential for reach and equity. The review underscores the need for adequately powered trials with prospectively measured falls as a primary outcome.
- Taylor ME et al. Implementation of a digital exercise programme in health services to prevent falls in older people. Age Ageing 2024;53(8):afae173.
  - ○ This study reports the real-world implementation of a digitally delivered exercise program within health services, moving beyond efficacy to the practical enablers and barriers of embedding digital falls prevention exercise into routine care, supporting its readiness for clinical practice.
- Dawson R, et al. The Telephysiotherapy for Older People (TOP-UP) program for improving mobility in people receiving aged care: a hybrid type 1 effectiveness-implementation randomised controlled trial. Med J Aust 2025;223(4):205–13.
  - ○ This hybrid type 1 effectiveness-implementation RCT evaluates telehealth-delivered physiotherapy for mobility in aged care recipients, supporting that telehealth-delivered exercise extends digital falls-prevention reach to higher-need, harder-to-access populations while surfacing the real-world implementation barriers (workforce, uptake).
- Eost-Telling C et al. Exergaming interventions for preventing falls and injurious falls in older people: systematic review and meta-analysis of randomised controlled trials. JMIR Aging 2026. https://doi.org/10.2196/89807
  - ○ This systematic review and meta-analysis provides a synthesis of exergaming as a falls prevention strategy, pooling evidence from nine randomised controlled trials involving 1,385 community-dwelling and residential older adults. These results show that falls reduction is achievable across different forms of digital exercise delivery.

## Data Availability

No datasets were generated or analysed during the current study.

## Declarations

### AI statement

Used Grammarly
